# Practice-Based Evidence to Support Return to Work in Cancer Patients

**DOI:** 10.3389/fresc.2022.819369

**Published:** 2022-07-07

**Authors:** Huget Désiron, Berthold Simons, Annemie Spooren, Stéphane Camut, Dominique Van de Velde, Thomas Otte, Théo Brunois, Kirsten Van Kelst, Lode Godderis

**Affiliations:** ^1^CEO ACT-Desiron BV, Hasselt, Belgium; ^2^Research Group Environment and Health, Department Public Health and Primary Care KU Leuven University, Leuven, Belgium; ^3^Research Group Innovation in Health Care, University College PXL, Hasselt, Belgium; ^4^Collège d'Ergothérapie de Bruxelles (CEBxl), Brussels, Belgium; ^5^Faculty of Medicine and Health Sciences, Department Occupational Therapy, Ghent, Belgium; ^6^Service Research and Quality, National Institute for Health and Disability Insurance, Brussels, Belgium; ^7^CEO IDEWE, Leuven, Belgium

**Keywords:** return to work, hospital-based support, cancer, evidence-based practice, practice-based evidence

## Abstract

**Background:**

International research emphasizes the importance of providing early, hospital-based support in return to work (RTW) for cancer patients. Even though oncology health professionals are aware of the scientific evidence, it remains unclear whether they implement this knowledge in current practice. This paper presents the knowledge and viewpoints of health care professionals (HCPs) on their potential role in their patients' RTW process.

**Methods:**

Semi-structured interviews with oncology HCPs were used to describe current practice. Results of these interviews served as input for focus group discussions with managers in oncology hospitals, which led to an agreement on of best practice.

**Results:**

This research had the participation of 75% of Belgian institutions involved in oncology health care services. Five themes were identified that influence care providers and staff to implement scientific evidence on RTW in cancer patients: (1) Opinions on the role that care institutions can take in RTW support; (2) Current content of RTW support during oncology care; (3) Scientific bases; (4) Barriers and success factors; and (5) Legislation and regulations. The key elements of the best practice included a generic approach adapted to the needs of the cancer patient supported by a RTW coordinator.

**Conclusions:**

Health care providers include RTW support in their current care, but in very varied ways. They follow a process that starts with setting the indication (meaning the identification of patients for whom the provision of work-related care would be useful) and ends with a clear objective agreed upon by HCPs and the patient. We recommend that specific points of interest be included in regulation at both the patient and hospital levels.

## Introduction

The recognition of cancer as a chronic disease is associated with the need to develop a corresponding approach to the long-term recovery process (1–4).

Globally, 5–year survival rates have increased, which is confirmed by the decrease in mortality rate over the past 20 years (5, 6). Belgian patients have a relatively good prognosis, with 5–year survival rates of more than 85% and a 10-year survival rate of more than 75% (1, 7, 8).

For cancer patients of working age, this implies that RTW support deserves to be implemented as part of the care provision. More than 40% of BC survivors do not succeed in resuming work (9–13). For the other 60 %, maintaining labor participation remains far from easy and may lead to job loss (14–17). Pauwels et al. underpin BC patients' needs for support regarding return to work (RTW) and indicate that, following patients' and caregivers opinions, those needs are insufficiently met (13). Patients' needs for RTW support should be addressed and integrated in healthcare services early in the treatment process (11, 13, 18–27). Research provides insight into the needs of cancer patients and the extent to which these – with regard to labor participation – are currently unanswered (3, 28–31). The literature also makes it clear that maintaining employment/resumption of work is an important element in the lives of working cancer patients (14–19) and that it is advisable to implement this in the provision of care (32–36).

The available scientific evidence on this subject seems to be insufficiently implemented in practice, creating a gap between “evidence-based practice” and current care practice. The concerns about the relevance of scientific research to practitioners in routine clinical settings motivate enhancing treatment quality takes a quite different form, namely practice-based evidence (37). Despite this trend in oncology health care, some efforts have been made to pay attention to RTW, with a certain level of ‘practice-based evidence' as results of those efforts. As a consequence, this way of implementing available scientific evidence appears to create a gap between evidence-based practice on the one hand and (experience-based) practice-based evidence on the other (38–42).

By examining the opinions and experiences of HCPs in this field, the primary objective of this study is to investigate the extent to which Belgian oncology caregivers include RTW as part of hospital care for cancer patients of working age. The second objective is to determine whether specific hospital-based guidelines could be beneficial for HCPs on how they can contribute to early support for RTW in cancer patients. Such guidelines could facilitate the process of RTW for individual cancer patients.

This study is structured to address the following research questions:

- What (science-based) approach is used to support RTW by HCPs in oncology care?
- What are the facilitators and barriers that affect success in support of RTW within the chosen approach?
- What do HCPs see as “hospital-based best practice” to provide support for cancer patients' RTW?

## Methods

This study, with its grounded theory framework, follows a quality characterization structure as this allows for the detection of non-group or situation-specific patterns and a detailed understanding of the practice-based perspectives of occupationally active cancer care providers and patients (42–46).

**Figure 1:**
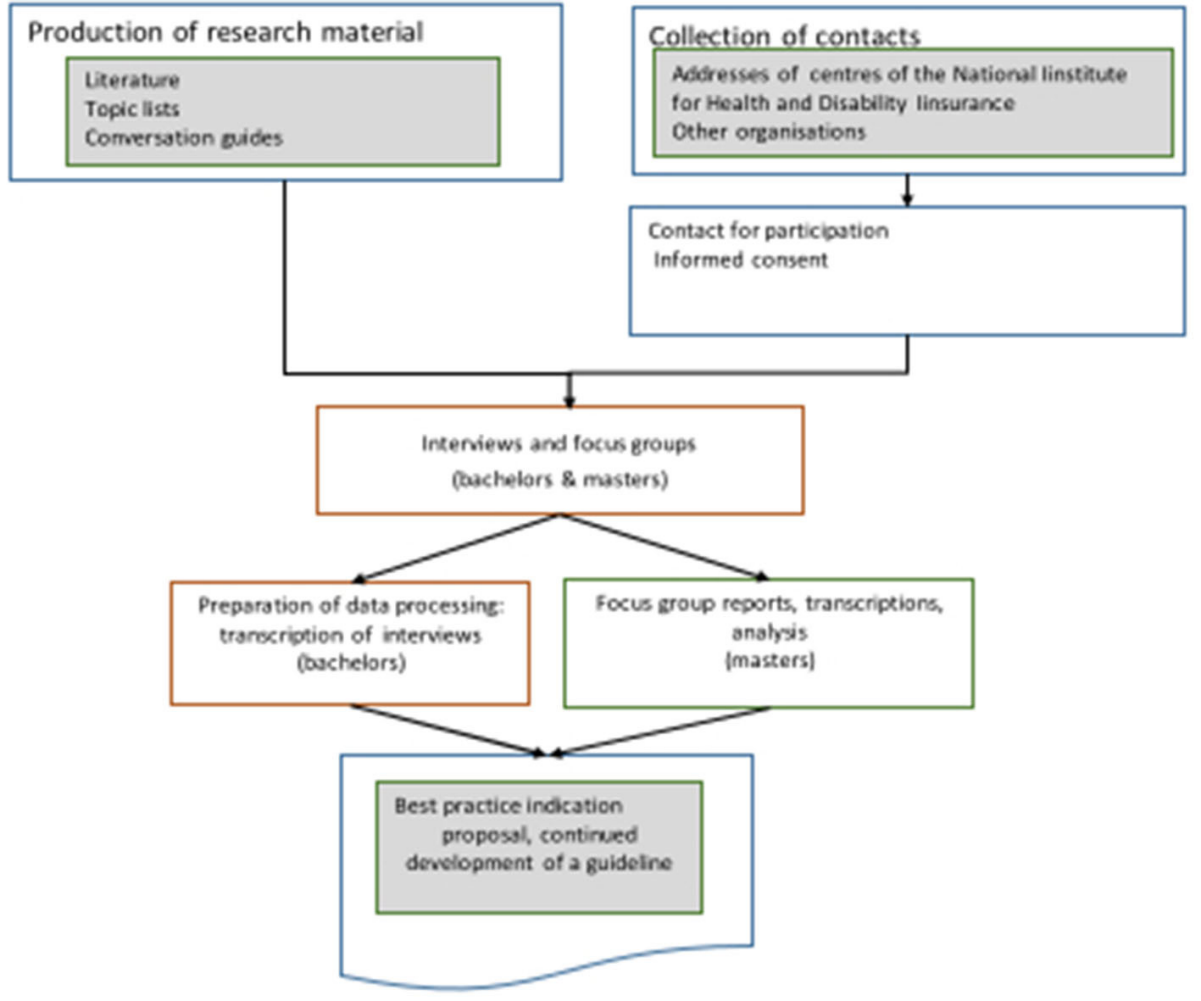
Practice-based evidence: research design.

This qualitative characterization study was designed using semi-structured interviews and focus group discussions. The study was carried out in collaboration with the Bachelor of Occupational Therapy courses (NL: university college PXL Hasselt/ Belgium; FR: university college CeBxl, Brussels/Belgium) and the master's course in occupational sciences (UGent/Belgium). Figure 1 visualizes the design of the research project.

Oncology services' websites, telephone contacts and email communications were used to recruit participants for the semi-structured interviews. These participants were care providers directly involved in the care of cancer patients (doctors, nurses, care coordinators, physiotherapists, occupational therapists, social workers, psychologists, onco-coaches, etc.).

When preparing the topic list for the interviews (see additional material for the interview topic list), the research group also took into account reflections obtained during the telephone recruitment from people who expressed that they did not see any use in including RTW support into the care programme and that they therefore did not wish to participate in this study. This recruitment procedure allowed the researchers to gain insight into which of the contacted HCPs provide hospital-based support for RTW in cancer patients in the hospital where they work. It also provided brief feedback on what approach is offered when the response was affirmative and, if the answer was negative, why certain professionals choose not to offer RTW support.

The interviews were carried out at the participants' workplace by specially trained pre-graduate occupational therapy students, who also transcribed the recordings. Analysis of the material was conducted by the researchers (B.S. and H.D), then supervised by the co-authors during regular research meetings. The interview guide that was used to structure the four focus group discussions was developed based on the analysis of the interviews (see additional material for the focus group guideline). This was carried out by the researchers with the assistance of a student preparing her final paper for a master's in occupational science.

The meetings for the focus groups took place in regional venues to avoid long travel times for the participants. The researchers took the role of moderators, while the collaborating student and the co-authors were responsible for written record keeping.

For both sets of data, NVIVO 12 software was used for encoding. Following the reasoning of ‘grounded theory', a code tree was developed by the researchers as the start of an open coding process. The concepts that were derived from the data were discussed with the co-authors and the participating students. They also collaborated in further analysis of the data using axial coding.

In addition to answering the study questions, the results of the analysis were used to make recommendations for implementing the findings and to develop proposals for a follow-up study targeting the development of a practice-based guideline.

## Results

### Recruitment

Seventy-four HCPs were willing to participate in the semi-structured interviews that took place at their workplace. The disciplines that participated in the research (interviews and focus group discussion) and the numbers per discipline are given in Table 1.

**Table 1:** Number of participants in semi-structured interviews and focus groups by discipline (ordered by location of the focus-group discussion).

**Discipline**	**Interviews**	**Focus groups**				
		**Brussels**	**Ghent**	**Namur**	**Leuven**	**Total FG**
Doctors	**13**	3	1	4		**8**
Oncology care coordinators	**17**	1	2	2	2	**7**
Occupational therapists	**4**					**0**
Physiotherapists	**3**	1				**1**
Social workers	**14**	1	3	3	5	**12**
Psychologists	**7**	1				**1**
Nursing staff	**9**		1	2	3	**6**
Others^*^	**7**	2	2		1	**5**
Total	**74**	**9**	**9**	**11**	**11**	**40**

* *FG, Focus group*.

### Qualitative Analysis

The analysis of the results was carried out on the records kept during the recruitment phase, ad verbatim transcripts of the semi-structured interviews, and ad verbatim transcripts of the focus group discussions. In total, 40 people participated in the focus group discussions.

**Analysis of the notes kept during the recruitment** made clear that a large majority of the care providers contacted for recruitment (more than 70%, or 122 people) believed that support for RTW should indeed be on the agenda, although they had not yet undertaken any initiatives as they were not sure how to address the matter. Ten per cent of the contacted HCPs believed that the care institution plays a real role in RTW and, as a result of this belief, were developing initiatives in this area. Thirty-five people from the contacted HCPs were convinced that promoting RTW support in the hospital setting is not one of the objectives or roles of a hospital. In the following text, the “italic” written text refers to input by participants of the focus group members.

#### Current Role of the Care Institution in Supporting People in Work or in Returning to Work

There was a broad consensus that a hospital can/should play a certain role in cancer patients' RTW. This consensus provided the impetus for a reflection, with the participants, on the current interpretation of the care offer. Providing information to patients on RTW seems to be a significant and frequently applied part of care. The participants made it clear that they consider broad dissemination of information to be important. This is usually done in individual consultations and/or by encouraging patients to participate in information sessions at group level but a more multidisciplinary approach is estimated to be beneficial: “*My ideal is a meeting, a multidisciplinary consultation of all the stakeholders at different times of the care to take stock, but this is the ideal world”* (FF2, 316-318).

#### Scientific Basis for the Information

In general, participants made little or no reference to the scientific evidence available to support the current method. However, they made reference to instruments used in the approach they followed., though it was not clear on what basis these instruments were selected.

#### Facilitators and Barriers in Practice

Concrete experiences from the participants' practice as well as their opinions and convictions on different points that they considered significant were discussed. As explained below, participants perceived several elements simultaneously as an obstacle and a success factor.

#### Lack of Knowledge

The participants were aware that they lacked knowledge about the legal and regulatory framework for occupational reintegration. This lack of up-to-date knowledge is particularly frustrating for the social workers but also for the other participants, and it has a negative impact on the quality of care they can provide. It was also mentioned several times that the lack of knowledge about the competences of other professionals led to a form of compartmentalization.

#### Presence of a Specific ‘Work Specialist' in the Team

Although the analysis reveals the need to assign tasks to care providers who have specific competences in a field, it turns out that participants also consider it important to be able to assign the topic of “work” to a specific care professional who can then be responsible for coordination: *He's someone who … brings together the elements of the file, there is the bridge, and there is the implementation, and. By saying, trust, someone, a person of trust* (FF2, 263-265)

Being responsible for this part of the care, this provider could then also refer to other team members for the treatment of specific points in the RTW pathway. However, the analysis also reveals a wide range of opinions as to which discipline within the team could or should be designated for this task.

#### Common Plan and Tailored Support

Participants agreed that a targeted approach to RTW support for occupationally active cancer patients should follow a stepwise plan that is the same for all patients as they progress in their recovery. There was also relative unanimity that such a “common step plan” must allow plenty of space for “tailored work” This should allow for different stages of the common plan to be carried out according to the patient's individual situation.

Participants who have experience with a concrete working method that has been in place in their hospital for some time note that some form of structure that sets out a modus operandi within the team is essential: “*But I think f… with these patients, we have to take it from the beginning. And one day you go back to work, one day you are expected to return to your workplace.”* (FN, 356-358)

Input from other participants also revealed that a structural approach that takes multi-disciplinarity into account is considered a success factor.

#### Case Management

There was a lot of vagueness about the criteria to be used to set the indication, who should take the final decision, who should monitor the follow-up and how this should be put into practice. There are also differences in opinion regarding the point at which such an indication is integrated into the patient's treatment pathway.

On the one hand, it is important that all stakeholders, including the patient, personal and professional network, health care providers and others, adopt the same point of view as soon as possible. On the other hand, there is a fear of frightening the patient by immediately broaching the subject of work in parallel with explanations about the therapeutic pathway. Providers feel that the ability to determine when it is still too early to mention RTW based on their assessment of the patient's condition is part of good care.

#### Factors of Influence

A clear distinction was made between when it is possible to talk about or provide information on RTW and when to start actions related to it. In both the interviews and the focus group discussions, providers indicated that a number of elements that influence the success of actions targeting RTW can be attributed to the attitudes, knowledge or beliefs of cancer patients. Providers particularly consider the following points:

- The Motivation of HCPs and Patients to Engage in RTW Therapies
- An understanding of HCPs' own abilities, which is (among other things) influenced by the importance of self-knowledge and self-confidence, to the extent that the wish to return to work corresponds to what is possible
- Practicalities Regarding Participation
- Information about possibilities, such as affordable and easily accessible care provision, information about and understanding of the legal measures that can be used
- The patient's relational network, where (among other things) there is social pressure or a lack of stimulation
- The extent to which patients can feel heard, which seems to be as much a barrier as a factor for success

#### Contact Between the Hospital and External Services

The particpants in this study are clearly convinced that collaboration with professionals outside the hospital is necessary to achieve good results: *And it is not only the hospital that must invest, it should be a collaboration between the hospital and external organizations* (IF5, 109-110).

The participants were also aware that maintaining contact with the working environment during the patient's recovery is a particularly strong success factor. At the same time, they indicated that they did not have a clear view on how they could play a role as care providers.

Regarding the necessary transmission of information from the care setting to the workplace, participants were very careful to respect the professional confidentiality and duty of discretion that is imposed on them.

Although participants reported that they rarely had the opportunity to make direct contact with the workplace of their cancer patients, there was disagreement about the extent to which this can (or cannot) be considered a component of care aimed at RTW. The analysis shows unanimity that the employer ultimately holds the key to achieving RTW.

The gradual implementation of RTW includes measures that–for both cancer patients and their employers–allow for the adaptation of the working environment, the reorganization of the content of tasks, the modification of working hours or the request for compensation for reduced performance, seem to be little known and are still rarely communicated as advice from the care providers to the patient.

#### Other Points of Hindrance for RTW

In addition, there are also several references to the problems that many cancer patients have with administration: “*But I think f… with these patients, we have to take it from the beginning and help them to be aware that one day they might go back to work, one day they are expected to return to their workplace*.” (FN, 356-358).

#### Preconditions

One of the aspects that participants with experience in supporting RTW found to be very relevant but which also often caused difficulties was the status under which patients were engaged. A wide range of different organizations in the field (e.g., home care, regional initiatives, patient advocacy organizations, self-help groups) also target “labor market participation” to a greater or lesser extent. Care providers find that consistency and collaboration in this area is far from optimal.

Moreover, from the hospital it is far from easy to get a good idea of who is best to contact in the company where their patient works. Presenting oneself as an oncology provider with a question about a worker has the consequence of informing the employer about the condition of the worker. This implies that the matter must be properly discussed with the patient beforehand and that an informed consent is signed. Without such consent, care providers cannot contact any other stakeholder.

#### Information on Best Practice and an Ideal Scenario

The responses of the participants in the interviews and focus group discussions on the ideal way of working were very diverse. However, two aspects emerge from the analysis of the responses:

- An ideal working method is an integrated process with good internal communication and a well-designed electronic patient file that is produced according to a general roadmap while also offering room for individual customization.
- A crucial role is assigned to a central figure who has a coordinating function and takes responsibility for the RTW process. This coordinator initiates the internal communication between care providers, manages the start and end time of the process, liaises with the workplace, leads the collaboration with intermediaries and is responsible for the management of measurement data regarding quality control and the effectiveness of RTW support.

“*In my ideal world, a kind of information desk would be associated with people who have received a specific education to be able to bring. …… from there we could get information about resumption of work and there could possibly still be a referral and where an extensive conversation is possible*.” (FN4, 445-451).

## Discussion

### Interpretation of the Concept of Role

**Figure 2:**
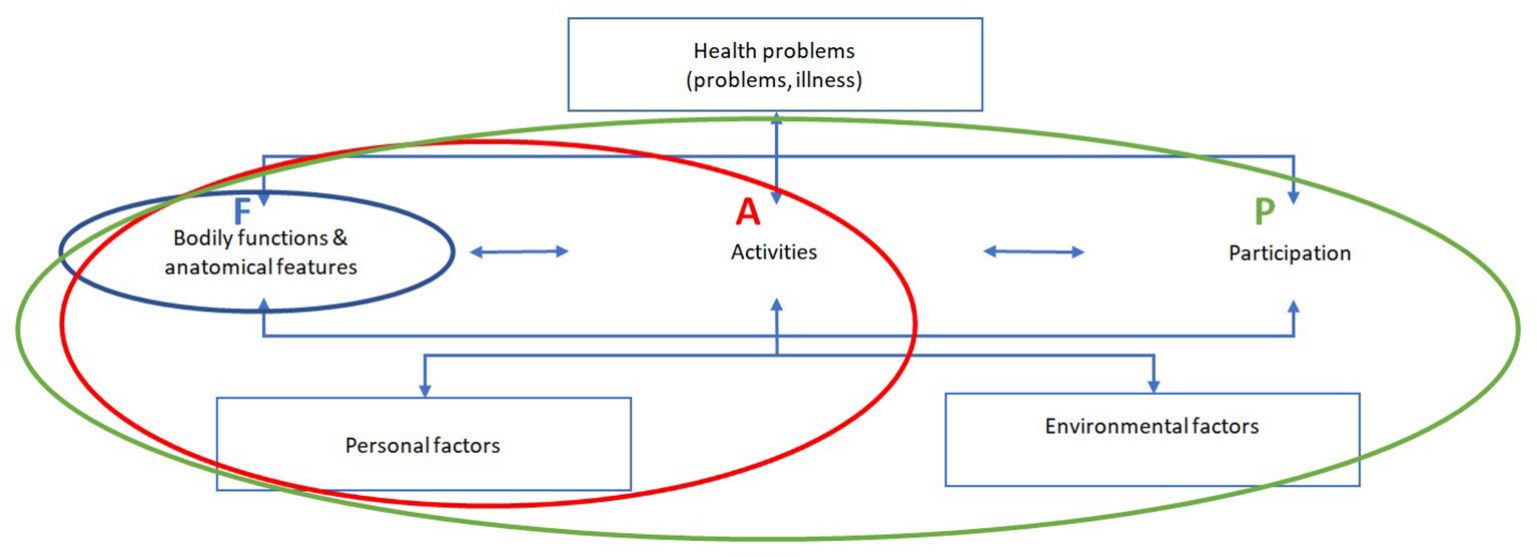
Differences in the purpose of care regarding RTW for cancer patients based on the ICF model.

Differences in participants' views on the extent to which cancer care providers or the organization can contribute to RTW can be indicated as follows (using the model of International Classification of functioning, disability and health; see Figure 2):

- Care is primarily aimed at restoring health. Restoration of quality of life is a different priority, and the focus is on the functional level, represented by **F** (**blue area)** in Figure 2.
- Care should have quality of life as its goal, which is reflected in the results of this study in the form of referral to additional care or services, such as psychological support, cosmetic advice and dietary advice. Special attention is given to the ability to function in daily life, represented in Figure 2 by component **A (red area)**.
- The participants demonstrate that the purpose of care for cancer patients must also consider the restoration of their involvement in society. The participants see this as an important component of the quality of life of these patients, as seen in component P (**green area**) in Figure 2.

### Interpretation of the Role of the Hospital and Care Providers

What the participants presented as the current approach in their hospital consists of a variety of in-hospital actions and collaboration with other stakeholders. Some of the approaches that appear in the analysis are ad hoc while others are part of a more formal process; most participants considered their approach as a work in progress.

**Figure 3:**
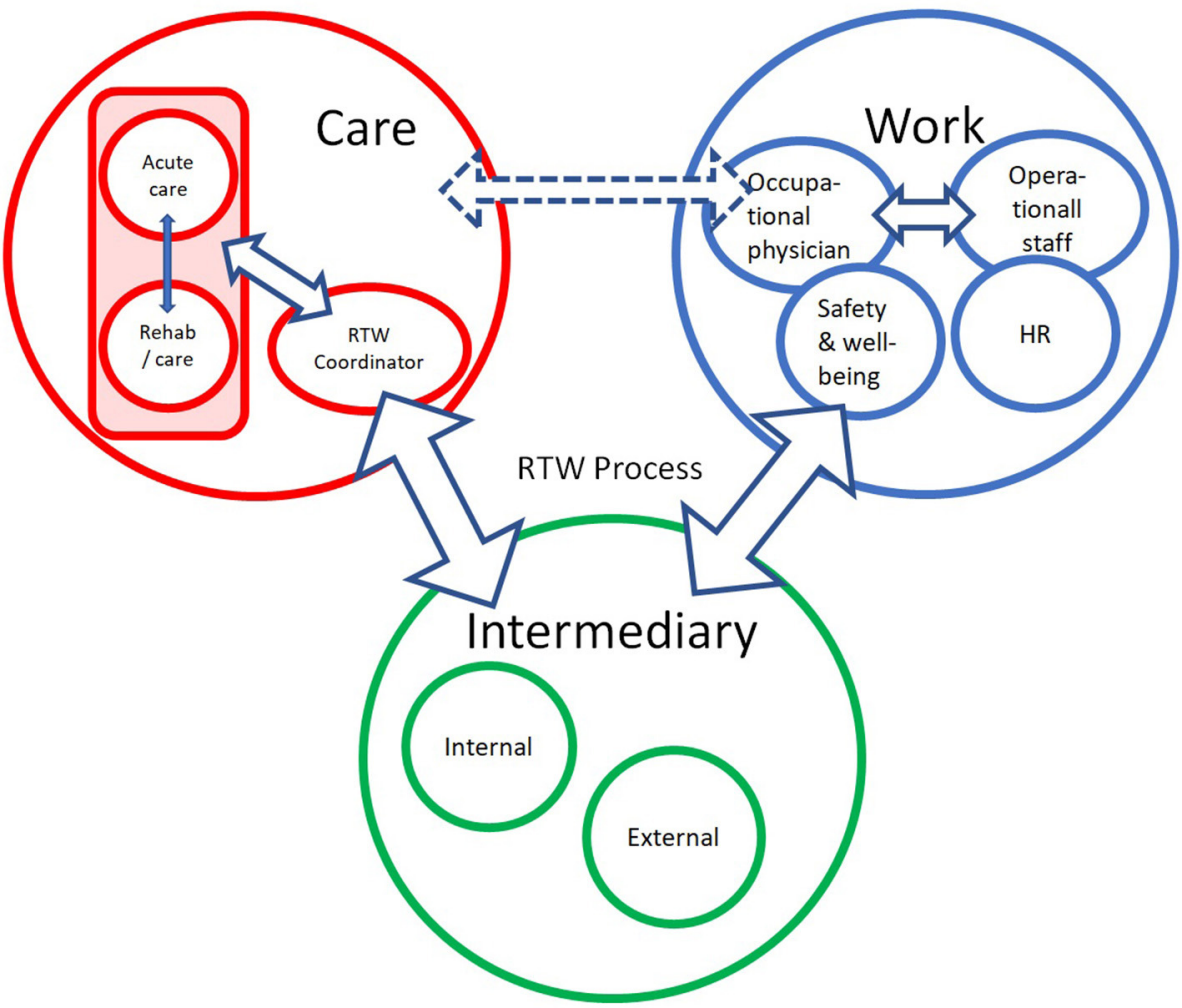
Schematic representation of the overview of the current meaning of the work-oriented approach. HR, Human Resources Department; Red circle indicates who within care is involved in RTW support; Blue circle refers to the stakeholders in the workplace; Green circle refers to specialists who play a role in the RTW process but are not part of the care team of the oncology or revalidation department.

The schematic visualization of their input in Figure 3 indicates that the current way of collaborating does not implement direct and well-organized communication between the care (red circle) and workplace (blue circle) settings. HCPs refer to this situation as complex and confusing. The shape and the marking of the arrows in Figure 3, which refer to the contacts and collaboration between different stakeholders, indicate the intensity of the interactions.

Almost all hospitals offer concrete information and assistance to cancer patients regarding administrative formalities (e.g., forms to fill in for social insurance matters or notes to establish sick-leave periods).

The people indicated in the red circle may be part of the service provision within the hospital or may, as external participants, offer input in supporting the RTW journey of individual cancer patients provided by patient organizations or fellow sufferers.

The focus on restoring physical capacities (e.g., fighting fatigue, increasing stamina, giving dietary advice) is given attention in some institutions, while others put more emphasis on emotional and psychological well-being. Very often, work-related issues are only put on the agenda after an explicit request from the patient.

In programmes where work is one of the components, the direct contribution of the hospital is limited, and external intermediaries are more often used. Direct contact with the employer, although desirable, rarely takes place.

### Indication

The indication^1^ to initiate or attend to work-related support is usually made in a non-systematic way and mainly on the basis of a direct request from cancer patients. The participants mainly refer to their own involvement in the patients' practical situation. However, the fact that they have too little information to gain a clear overview of the elements that are important in the complexity of the problem increases the risk of a mismatch.

### Best Practice

**Figure 4:**
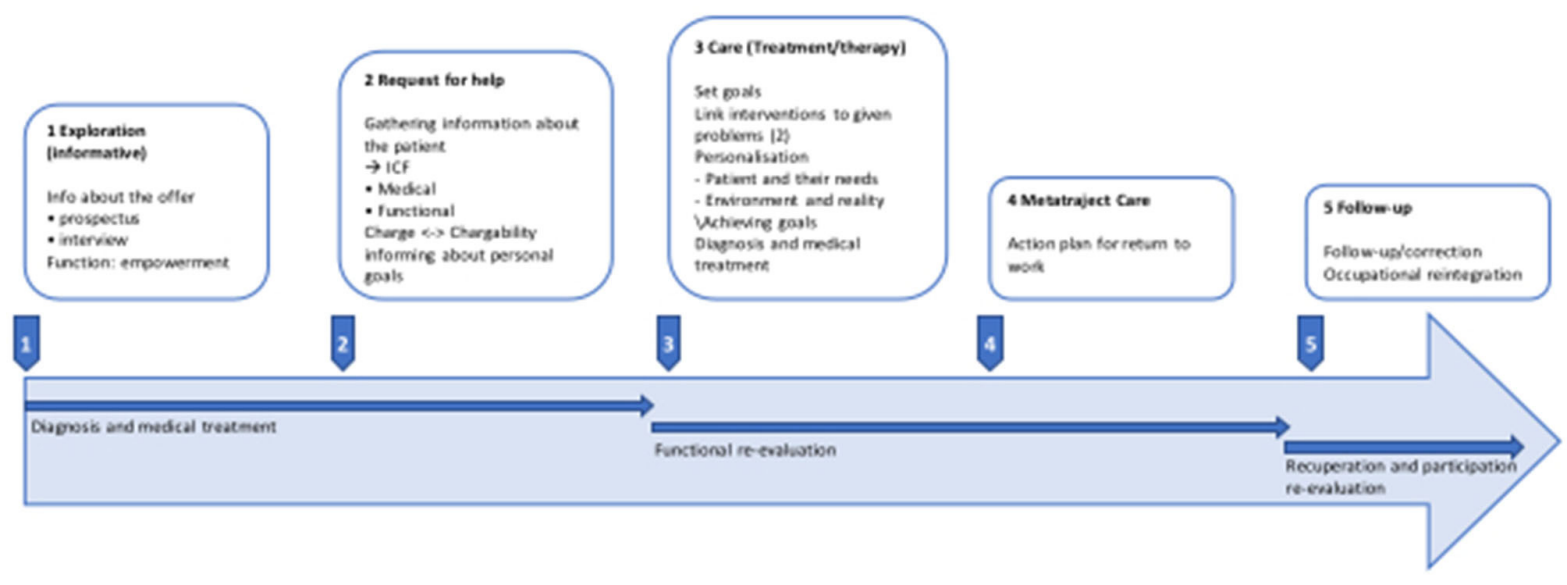
Step-by-step plan for work-oriented care during the cancer patient's care journey.

Innovative thinking about a more fitting approach results mainly in the need for more staff, resources and possibilities. The steps implemented by care providers in some hospitals to facilitate cancer patients' journey toward promoting RTW are shown in Figure 4.

Monitoring the maintenance of functional recovery (see also red circle of Figure 3) and attending to the balance between the person's capacity and the burden in the person's life seem to be essential to a successful RTW journey. These components are the basis for the development of work-oriented goals, which requires collaboration with all stakeholders.

It is these people who, during Phase 4, will shape the content of the RTW action plan and decide who will be involved and when. This should ensure that medical–functional problems are dealt with in a timely manner while avoiding any risk of dependency on the hospital.

The approach developed, implemented and by Désiron et al. was used in the KOTK-funded BRUG study project (47–50). It incorporates the above-mentioned elements and shows that the implementation of the listed components can offer significant benefits to oncology through attention to the restoration of participation.

Both parts of the study highlight the need to eliminate the disadvantages of the current shared responsibility, where it is unclear, for example, who does what and when, where the contributions of different disciplines overlap and where gaps exist. Clarity can be achieved by creating a coordinating function responsible for, among other things:

- Monitoring the RTW process
- Coordinating internal communication
- Drawing on the knowledge of others
- Ensuring that the necessary knowledge is available and up to date
- Putting together the RTW file
- Respecting privacy and medical confidentiality
- Providing functional information
- Managing administrative formalities
- Establishing contacts/collaboration with external parties (intermediaries and/or stakeholders).

The international literature on RTW support explores the possibility of utilizing different disciplines among care providers and the extent to which the RTW care needs of cancer patients are met (33, 49, 51–55). Although each of the care providers mentioned can make a relevant contribution, the literature shows that none of these disciplines has the full range of competences needed. In this study, care providers state that they assign a person to a coordinating function if they feel the need, while they refer directly to the need to prioritize the necessary time and resources for this purpose.

**Figure 5:**
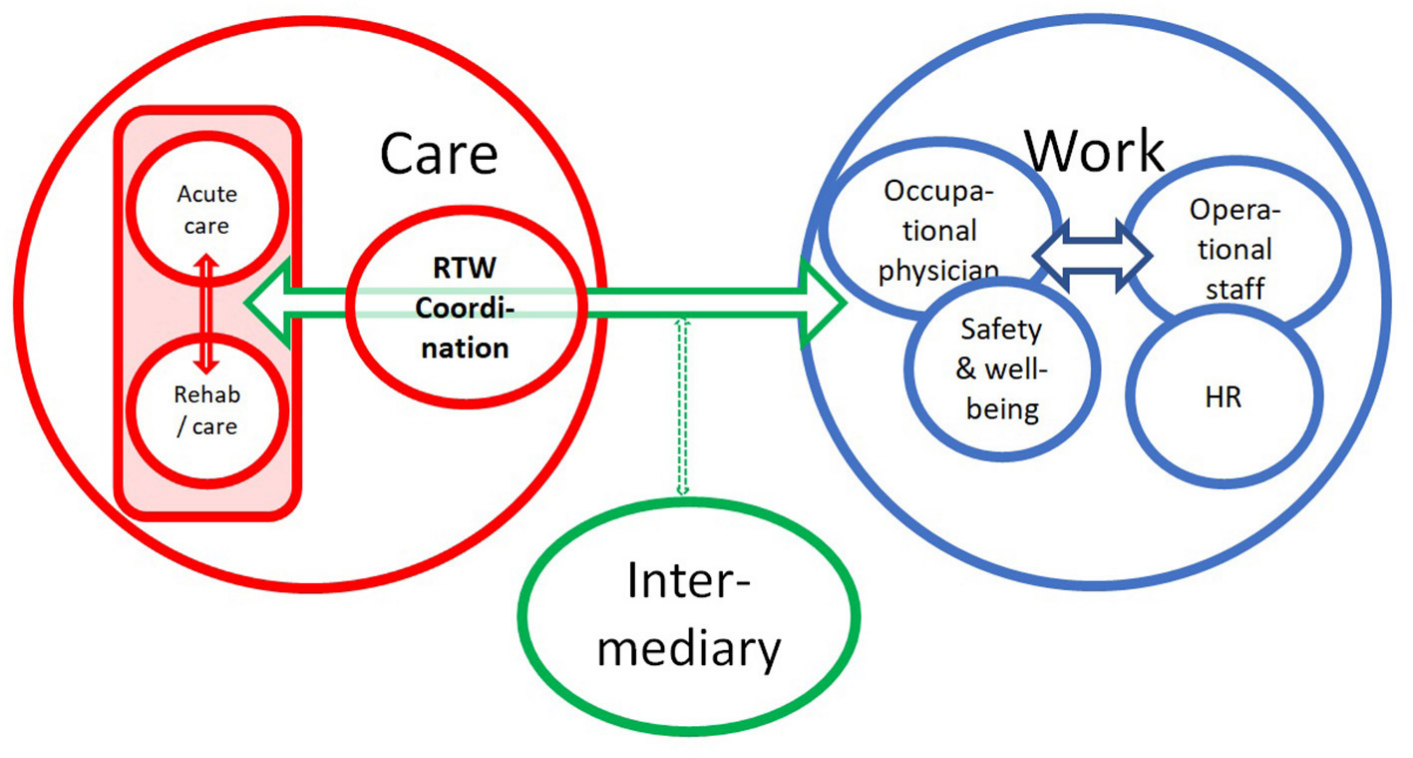
Schematic representation of best practice for the role of hospitals in RTW support for occupationally active cancer patients.

Based on the input by the HCPs about their current practice regarding their efforts in support of RTW (see Figure 3), Figure 5 represents the conclusions that emerged from discussion of these findings with the research group at the end of the analysis process. The figure visualizes an ideal scenario for fulfilling the role that hospitals could play regarding RTW support for cancer patients and will be used as basis for further development of the RTW guidelines, to which this study aims to contribute.

The RTW coordinator, who is a member of the team and as the person responsible for the patient's file has legal access to the entire patient file, is responsible for coordination and internal communication with the patient, their relatives and other members of the team (see red circle). Direct access is necessary to initiate smooth collaboration between the professionals involved in the shared confidential medical information. It also facilitates smooth and correct communication with professionals within the team who cannot legally have direct access to medical information.

The RTW coordinator assumes responsibility for the process and therefore supports the team members in their contribution according to their function. The coordinator is also the fixed point of contact for other workplace stakeholders (see blue circle) and is responsible for establishing contacts and consultations with intermediaries (see green circle), such as medical advisors for mutual societies or specific service providers.

The large green arrow indicates that direct communication with workplace stakeholders is a priority and that the involvement of intermediary partners in this area can make a significant contribution.

### Methodological Considerations

In absolute numbers, the number of participants (*n* = 103) in relation to the number of cancer care providers is not high, in contrast to the number of care institutions represented. Of the institutions providing oncology care in Belgium (85), more than 75% (*n* = 63) delegated one or more persons. The relative advantage of the telephone approach compared to email recruitment is that it provides a view on the reason for non-participation. The disadvantage, however, is that the discussions left virtually no opportunity for further exploration of the reasons given by these individuals for not attributing a role to hospitals in this matter.

The variety of disciplines among the participants in the interviews and focus group discussions gives an overview of the disciplines that currently engage with work as a component of care or are delegated to do so by their institution, at different levels of direct involvement. Though the topic of this research is supporting BC patients to maintain / regain occupation (in casu work), it is remarkable that so few occupational therapist were participating in this research project. This can be partially understood by the fact that – in Belgian oncology care – occupational therapy is not structurally prescribed, even though scientific research support the input of this paramedical discipline in many domains of onco-care (35, 47, 56–62). From the viewpoint of the “evidence based practice” that is presented in literature, the poor representation of occupational therapists might increase risk of selection bias. That might also be the case for all other participating disciplines but this qualitative research project did not focus on representativity of HCPs' disciplines. As stated in the introduction to this paper, the primary objective of this study is to investigate the extent to which Belgian oncology caregivers include RTW as part of hospital care for cancer patients of working age. The phased approach of this PBE-research project provides information on the state of play in the field through the interviews but also, through the focus groups. This gives an insight into the context and policy decisions taken by institutions and care providers regarding the provision of care (in which they indicate RTW might/should be integrated).

In line with indications by the participants in our research, Bilodeau et al. state that a RTW-intervention would need to focus on both creating the conditions to change practices in favor of the intervention and making the intervention an integral part of professional practices and the organization of existing services (63, 64).

Regarding the first research question of this PBE-project on having access to scientific literature, participants indicate that is very difficult (e.g., no online access, few opportunities to participate in congresses on RTW, not enough time to read an discuss scientific information,…). The participants' input aligns with the point of view of Gabbay et al., who state that practitioners in health care found their knowledge on more than scientific literatures' information: the use of their practical skills, soft skills, technical skills, illness scripts, heuristics, rules of thumb, embedded science, guidelines, peer values, institutional culture, roe models' behavior, local norms/routines, trainers'/teachers' norms and tacit and experiential knowledge (40).

## Conclusion

With focus on what (science-based) approach is used to support RTW by HCPs in oncology care, this project lead to the conclusion that in most care institutions, work is an issue that receives attention through very varied approaches, although in a small number of hospitals, the approach is systematic and structured. Although the input of participants aligns with evidence provided by literature, our result shows

There is also a great diversity in the provision and an equally great variation in the intensity of actions focused on work. The contribution of health care providers in supporting the re-establishment of work participation usually takes place when the patient asks concrete questions about it, and the contribution is currently mainly reflected in the efforts of social workers to inform their patients about the administrative formalities related to work incapacity and consequent benefits. This input is generally not based on a scientific model. Their visions of best practice provide a concrete form and content for actions targeting the cancer patient and solving organizational and administrative problems.

Following the input of the participants, facilitators and barriers that affect success in hospital based RTW support often form a clear ‘mirror image' of each other, e.g., knowledge is seen as a strong support, the lack of it is indicated to be an important barrier. The same “mirror image” is named for issues such as (lack of) presence of an “work specialist” in the team, common and patient-tailored support in the organization of the care and in the organization of work oriented services in the hospital, implementation of case-management, contact between hospital and external services that offer RTW support… Our results show that avoiding the barriers mentioned is very much congruent with the mentioning (by other participants) of success-factors.

HCPs see implementation of those success factors / avoiding the barriers mentioned as “hospital-based best practice” to provide support for cancer patients' RTW. More specific, this means:

- Monitoring the RTW process
- Drawing on the knowledge of others
- Ensuring that the necessary knowledge is available and up to date
- Putting together the RTW file
- Respecting privacy and medical confidentiality
- Providing functional information
- Managing administrative formalities
- Establishing contacts/collaboration with external parties (intermediaries and/or stakeholders

Recommendations from this study to both policy-makers and practice therefore relate to the development of a concrete and usable guideline that provides clear information on the following topics:

- The process that can be used for patients to explore and realize their chances of returning to work.
- Essential elements for organizing the optimal integration of care provision into the hospital's operation. A key point highlighted was the need for organized coordination of support for RTW, which could possibly be organized at the hospital level (i.e. across pathologies).

## Data Availability Statement

The datasets presented in this article are not readily available because application was not agreed upon in the informed concent. Requests to access the datasets should be directed to huget@act-desiron.be.

## Ethics Statement

The studies involving human participants were reviewed and approved by Sociaalmaatschappelijke Ethische Commissie (SMEC) of KULeuven, Belgium on 04/05/2018 (code: G-2018 04 1218). The participants (all healthcare professionals) provided oral and written informed consent to participate in this study.

## Author Contributions

HD, BS, and LG contributed to conception and design of the study. All authors participated in the elaboration of the study and contributed to manuscript revision, read, and approved the submitted version.

## Funding

This study was funded by the Belgian National Institute for Health and Disability Insurance.

## Conflict of Interest

LG is employed by IDEWE but no conflict of interest is to be reported. The remaining authors declare that the research was conducted in the absence of any commercial or financial relationships that could be construed as a potential conflict of interest.

## Publisher's Note

All claims expressed in this article are solely those of the authors and do not necessarily represent those of their affiliated organizations, or those of the publisher, the editors and the reviewers. Any product that may be evaluated in this article, or claim that may be made by its manufacturer, is not guaranteed or endorsed by the publisher.

## Footnotes

^1^The term “indication” here refers to the identification of patients for whom the provision of work-related care would be useful. It is important to be able to offer patients for whom this is not yet on the agenda (e.g. those who are willing and able to consider a return to work of their own free will) adequate and tailored care based on rigorous follow-up.
